# Robust and Intensity-Dependent Synaptic Inhibition Underlies the Generation of Non-monotonic Neurons in the Mouse Inferior Colliculus

**DOI:** 10.3389/fncel.2019.00131

**Published:** 2019-04-04

**Authors:** Yun Liu, Guodong Zhang, Haipeng Yu, He Li, Jinxing Wei, Zhongju Xiao

**Affiliations:** Department of Physiology, School of Basic Medical Sciences, Southern Medical University, Key Laboratory of Psychiatric Disorders of Guangdong Province, Guangdong-Hong Kong-Macao Greater Bay Area Center for Brain Science and Brain-Inspired Intelligence, Key Laboratory of Mental Health of the Ministry of Education, Guangzhou, China

**Keywords:** the central nucleus of the inferior colliculus, intensity-tuned neurons, non-monotonic neurons, monotonic neurons, *in vivo* whole-cell recording, synaptic currents

## Abstract

Intensity and frequency are the two main properties of sound. The non-monotonic neurons in the auditory system are thought to represent sound intensity. The central nucleus of the inferior colliculus (ICC), as an important information integration nucleus of the auditory system, is also involved in the processing of intensity encoding. Although previous researchers have hinted at the importance of inhibitory effects on the formation of non-monotonic neurons, the specific underlying synaptic mechanisms in the ICC are still unclear. Therefore, we applied the *in vivo* whole-cell voltage-clamp technique to record the excitatory and inhibitory postsynaptic currents (EPSCs and IPSCs) in the ICC neurons, and compared the effects of excitation and inhibition on the membrane potential outputs. We found that non-monotonic neuron responses could not only be inherited from the lower nucleus but also be created in the ICC. By integrating with a relatively weak IPSC, approximately 35% of the monotonic excitatory inputs remained in the ICC. In the remaining cases, monotonic excitatory inputs were reshaped into non-monotonic outputs by the dominating inhibition at high intensity, which also enhanced the non-monotonic nature of the non-monotonic excitatory inputs.

## Introduction

Intensity and frequency are two fundamental characteristics of an acoustic stimulus. The auditory system coding of sound intensity in people is not as well understood as its coding of frequency (Dean et al., 2005; Uppenkamp and Röhl, 2014). Neurons in the auditory system that differ from other sensory systems not only exhibit a monotonic change in stimulus intensity (the discharge rate of neurons increases with an increase in stimulus intensity) but also a non-monotonic change. That is, the discharge rate increases to a certain level and then decreases as the sound intensity increases. To date, in many animal species, non-monotonic neurons have been found in each nucleus of the ascending central auditory pathway, including the cochlear nucleus (CN; Ding and Voigt, 1997; Ding et al., 1999; Davis and Young, 2000), the inferior colliculus (IC; Aitkin, 1991; Ramachandran et al., 1999; Cabrera et al., 2013), the medial geniculate body (MGB; Aitkin and Webster, 1972; Rouiller et al., 1983; Rodrigues-Dagaeff et al., 1989), and the auditory cortex (AC; Schreiner et al., 1992; Barone et al., 1996; Polley et al., 2006). The non-monotonic intensity discharge function was considered to be a possible mechanism for coding intensity; therefore, the non-monotonic neurons can also be called intensity-selective neurons (Zhou et al., 2012). In a sound intensity discrimination experiment (Polley et al., 2004; Tan et al., 2007), the number of non-monotonic neurons in the AC of trained rats was increased, suggesting that non-monotonic neurons contribute to the recognition of acoustic sound. Because the intensity of a sound is often an important guide for behavior (Chen et al., 2012; Takeshima and Gyoba, 2013; Clemens et al., 2018) and non-monotonic neurons are rare in other sensory systems (Chapman et al., 2002; Peng and Van Essen, 2005; Peirce, 2007; Sofroniew et al., 2015), the underlying mechanisms of non-monotonic neurons in the auditory system have generated widespread interest.

There are few non-monotonic coding strategies in the auditory nerve (Kiang et al., 1965; Sachs and Abbas, 1974; Gifford and Guinan, 1983) that are only in the central auditory area. The percentage of non-monotonic neurons gradually increases along the auditory neuraxis from less than 15% in the CN (Davis et al., 1996; Navawongse and Voigt, 2009; Ma and Brenowitz, 2012; Zhou et al., 2012) to near 80% in the AC (Wu et al., 2006; Sadagopan and Wang, 2008; Watkins and Barbour, 2008). Therefore, the inhibition from the central nervous system is required for the formation of the non-monotonic intensity-response function. Non-monotonic neurons have been considered to be produced by a reduction in the response at high sound intensity upon the interaction of excitatory and inhibitory inputs (Sutter and Loftus, 2003). To better understand how integrating excitatory and inhibitory inputs produce non-monotonic neurons, whole-cell voltage-clamp is a useful technique that is able to examine sound-evoked synaptic inputs directly. In previous studies, in the AC (Wu et al., 2006; Tan et al., 2007), the unbalanced intensity tuning and temporal properties of excitatory and inhibitory inputs are the keys to the non-monotonic intensity-response function of neuronal firing. In this case, cortical intensity tuning is primarily inherited from its excitatory inputs, but the inhibitory inputs can enhance the intensity tuning. Using whole-cell voltage-clamp techniques in the CN Zhou et al. (2012), also revealed that the different intensity-tuning properties between excitation and inhibition determine the generation of non-monotonic neurons. There are two types of monotonic intensity responses in auditory nerve fibers: fast saturating and slow saturating. The DCN intensity-selective neurons receive fast-saturating excitation directly from auditory nerve afferents and slow-saturating inhibition from local inhibitory neurons. As a result, selective neurons can be created in the dorsal CN by differential synaptic intensity tuning. In the central nucleus of the ICC, non-monotonic neurons may also receive multiple forms of excitatory and inhibitory inputs according to previous observations by blocking the local inhibitory circuit (LeBeau et al., 2001; Sivaramakrishnan et al., 2004; Tang et al., 2008). It was said that the excitatory output in ICC could be changed to non-monotonic by integrating a temporally delayed inhibition or be maintained monotonicity by the GABAergic inputs in removing firing block. What’s more, the lateral inhibitory processes occur in the overlapped region of inhibition and excitation input and change frequency response area, indicating that the excitatory or the inhibitory domain may determine intensity tuned or not. Although these studies recognize the importance of an inhibitory mechanism to generate non-monotonic neurons in the ICC, the specific excitatory and inhibitory interrelationships and forms of integration remain unknown. Because the ICC is the first integration center of the auditory system, studying the non-monotonic transmutation in the ICC is of great significance.

We, therefore, performed *in vivo* whole-cell recordings to directly examine the synaptic excitation and inhibition of ICC neurons in pentobarbital-anesthetized mice. Our data indicated that the ICC neurons receive different intensity-tuning properties between excitatory and inhibitory inputs. Compared with the number of monotonic excitatory synaptic inputs, there are more monotonic inhibitory inputs on ICC neurons. This increase in monotonic inhibition can improve the inherited non-monotonic intensity tuning. In addition, the synaptic inhibition imbalance can also create non-monotonic neurons in the ICC. This study may help us understand the role of central inhibition in the creation of non-monotonic neurons.

## Materials and Methods

### General

All animal experiments in this study were approved by the Animal Care and Use Committee of Southern Medical University. Seventy-nine female C57BL/6J mice (4–6 weeks, 14–20 g, housed under a 12 h light/dark cycle) obtained from the Experiment Animal Center of Southern Medical University, Guangzhou, China, were used in this study. All efforts were made to minimize the number of animals used and their suffering.

### Animal Preparation and Mapping of the ICC

All mice prepared for electrophysiological recording experiments were subjected to surgery following our previously reported methods (Tan et al., 2008; Huang et al., 2015; Wei et al., 2018). After atropine sulfate (0.25 mg/kg, Nandao, Hainan, China) was injected subcutaneously to reduce tracheal mucous secretion, mice were anesthetized with sodium pentobarbital (60–70 mg/kg, i.p.). During the surgical preparation, the pedal withdrawal reflex of the animal was checked, and anesthesia was maintained by supplemental doses of sodium pentobarbital (13 mg/kg). Then, the scalp was removed, and a 1.5 cm nail was fixed to each mouse skull surface with dental cement. In addition, a small craniotomy was made to open a window (0.5 × 0.5 mm^2^) over the left ICC (according to the atlas for the mouse brain: −5.2 mm from Bregma and 1 mm lateral to the midline) without removing the dura. The window was covered with Vaseline, and the wound was covered with lidocaine hydrochloride (Suicheng, Zhengzhou, China) as a local anesthetic. After surgery, the mouse was returned to a cage for recovery with food and water *ad libitum*. To prevent infection, antibiotic ointment was applied to the surgical wound once a day.

### *In vivo* Whole-Cell and Loose-Patch Recordings

After 3 or 4 days for recovery, the mouse was re-anesthetized with pentobarbital for recording. The head of the animal was immobilized by insertion of the mounted nail into a small metal rod and its fixation with screws. After the Vaseline and dura were removed, a glass micropipette (tip diameter: approximately 1.5 μm, impedance: 4–7 MΩ) was inserted into the ICC region vertically to the brain surface with a micromanipulator (Siskiyou Inc, Grants Pass, OR, USA). For *in vivo* loose-patch recordings, the pipette was filled with artificial cerebrospinal fluid (ACSF; in mM: 124 NaCl, 2.5 KCl, 25 NaHCO_3_, 2 CaCl_2_, 1 MgCl_2_, 1.23 NaH_2_PO_4_, 20 glucose, pH = 7.28), while for whole-cell recordings, the pipette solution contained (in mM) 125 Cs-gluconate, 5 TEA-Cl, 4 MgATP, 0.3 GTP, 8 phosphocreatine, 10 HEPES, 10 EGTA, 2 CsCl, and 1 QX-314 (pH = 7.25). A reference electrode was placed under the frontal bone. When the pipette patched a neuron with a loose seal (0.2–1 MΩ), the recording was in cell-attached mode. Once the pipette patched a neuron with a giga-ohm seal, suction was applied to the pipette to perform whole-cell recordings (Xiong et al., 2013; Wang et al., 2017; Wei et al., 2018).

Cell-attached and whole-cell current-clamp recordings were performed using a MultiClamp 700B amplifier (Molecular Devices, Union City, CA, USA) in current-clamp mode and voltage-clamp mode, respectively. For voltage-clamp recordings, the whole-cell capacitance and pipette capacitance were completely compensated, and the initial series resistance (20–40 MΩ) was compensated for 50%–60% to achieve an effective series resistance of 10–20 MΩ. Signals were filtered at 5 kHz and sampled at 10 kHz using Digidata 1440A digitizer (Molecular Devices, Union City, CA, USA). Only neurons with resting membrane potentials lower than −45 mV and stable series resistance were used for further analysis of whole-cell recordings. The recording session lasted for approximately 3–4 h. All experiments were carried out in a soundproof room (24–26°C), and the animal’s body temperature was continuously monitored and maintained at 37°C using a heating pad with a feedback controller. After experiments, each mouse was euthanized with an overdose of sodium pentobarbital (120 mg/kg, i.p.).

### Acoustic Signal Generation

Acoustic stimulation was similar to that described in our previous article (Tang et al., 2008; Wei et al., 2018). Tone bursts (50 ms duration with 5 ms rise/fall time) of frequencies (2–64 kHz, at 0.1 octave interval) and intensities (0–70 dB, in 10 dB step) were presented to the contralateral ear in a randomized sequence (F-A scan). The acoustic stimuli were generated and delivered by Tucker-Davis Technologies System 3 (TDT 3, Tucker-Davis Technologies, Alachua, FL, USA). The sinusoidal signals were synthesized from RX6 and amplified by an electrostatic speaker driver (ED1). The intensities were controlled by PA5 (a programmable attenuator). Sounds were delivered to the mouse’s ears by two closed loudspeakers (EC1, frequency range 0.1–100 kHz) through small metal tubes. The tip of the metal tube was inserted into the external auditory meatus. The loudspeaker was calibrated with 1/8 and 1/4 inch microphones and an amplifier (Brüel and Kjaer 4138, 4135 and 2610, Naerum, Denmark). The amplitude of pure tone bursts was depicted as the sound pressure level (SPL, referred to 20 μPa). The parameters of sound (frequency, intensity, duration, rise/fall time) were controlled by Brain Ware software (Version 9.21, Tucker-Davis Technologies, Alachua, FL, USA) through a computer.

### Data Analysis

Spike tonal receptive fields (TRFs) were mapped for at least three repetitions, and synaptic current TRFs were mapped for one to two repetitions. Membrane potential TRFs were mapped for one to three repetitions. Tone-driven spikes were counted within a 0–100 ms time window after the tone onset from the post-stimulus spike time histogram (PSTH). The average number of evoked spikes for each tone was used for plotting the spike TRF. The characteristic frequency (CF) for the spike TRF was defined as the logarithmic center of the responding frequency range at the intensity threshold. Synaptic current and membrane potential response traces evoked by the same test stimuli were averaged. The evoked response onset was identified as the average trace higher than the average baseline activity by 2 SDs of the baseline fluctuation.

### Predicted Membrane Potential

The method of calculating the predicted membrane potential *V*_m_ followed the equation of previous articles (Wu et al., 2006; Tan et al., 2007; Zhou et al., 2012; Li et al., 2018).

$$V_{m}\left(t+\text{d}t\right)\;=-\frac{\text{d}t}{C}[G_{e}\left(t\right)*\left(V_{m}\left(t\right)-E_{e}\right)+G_{i}\left(t\right)*\left(V_{m}\left(t\right)-E_{i}\right)+G_{r}\left(V_{m}\left(t\right)-E_{r}\right)]+V_{m}\left(t\right)$$

where *V*_m_ is the membrane potential at time *t*; *C* is the whole-cell capacitance; *G*_r_ is the resting leakage conductance; *E*_r_ is the resting membrane potential (−50 mV); *G*_e_(*t*) is the excitatory synaptic conductance; *G*_i_(*t*) is the inhibitory synaptic conductance; and *E*_e_ and *E*_i_ are the reversal potentials of the excitatory and inhibitory synaptic conductance, respectively. *C* was derived from the whole-cell capacitance compensation procedure, and *G*_r_ was calculated using *G*_r_ = C*G_m_/*C*_m_, where *G*_m_, the specific membrane conductance, is 2 × 10^−5^ S/cm^2^, and *C*_m_, the specific membrane capacitance, is 1 × 10^−6^ F/cm^2^ (Hine, 1993; Stuart and Spruston, 1998). *G*_e_(*t*) and *G*_i_(*t*) were derived using Δ*I* = *G*_e_(*V* − *E*_e_) + *G*_i_(*V* − *E*_i_). Δ*I* is the amplitude of the synaptic current response at any time point after subtraction of the baseline current; *V* is the holding voltage; and *E*_e_ (0 mV) and *E*_i_ (−70 mV) are the reversal potentials of glutamatergic and GABAergic (Cl^−^) currents. By holding the recorded cell at these two different voltages, *G*_e_ and *G*_i_ were resolved from the equation.

### Statistics

On the basis of the acquired datasets, the monotonicity index (MI) was defined as the ratio of the spike counts (or the current amplitude) at the best amplitude (BA, the amplitude of the acoustic stimulus that evoked the most spikes) to that at the highest amplitude (HA). The values of relevant parameters were calculated using Excel 2016 (Microsoft). All statistical analyses were performed using SPSS statistics 19 (IBM). Two sample *t*-tests were used for two-group comparisons. For two sets of categorical counts, the chi-square test was applied to test significance. The F-test was used to evaluate the variance equivalence. Significance was defined as *p* < 0.05. Data fitting and plotting were carried out in OriginPro 8 (OriginLab). The results are presented as the mean ± SEM, if not otherwise specified.

## Results

### Non-monotonic Neurons in the Mouse ICC

**Figure 1:**
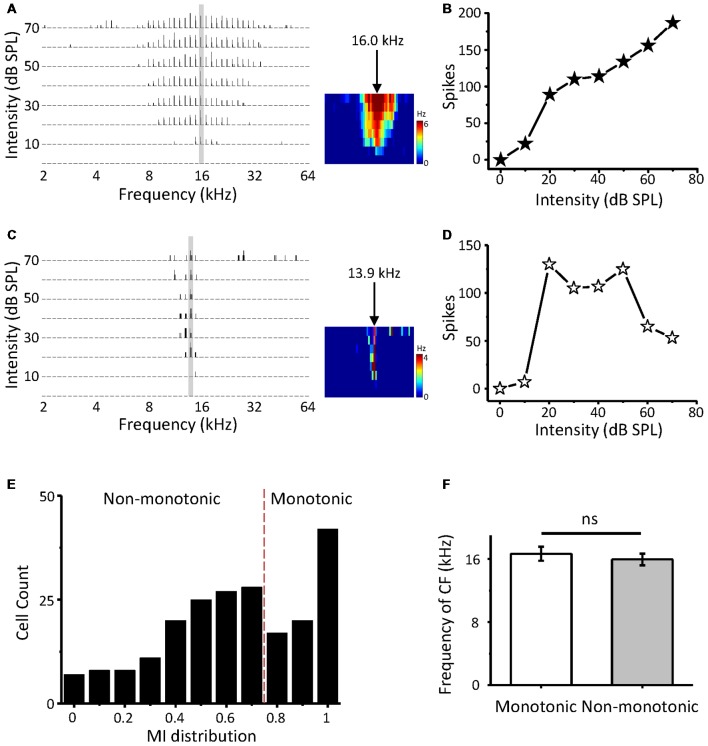
Intensity response properties of mouse inferior colliculus (ICC) neurons. **(A)** Tonal receptive field of spike responses (spike TRF) of an example ICC neuron, examined by loose-patch recording. Left, each small trace is a post-stimulus spike time histogram (PSTH) evoked by 50 ms tone bursts of a particular frequency and intensity combination (five repetitions). Right, color map depicts the average spike TRF within the 50 ms window. The arrow indicates the characteristic frequency (CF). **(B)** Plot of spike counts evoked by the CF (30 repetitions) as a monotonic function of intensity at the neuron in **(A)**. **(C,D)** An example non-monotonic neuron in the ICC. Data are presented in the same way as in **(A,B)**. **(E)** Distribution of the monotonicity index (MI) of ICC neurons (*n* = 213). **(F)** Comparison of CFs between monotonic and non-monotonic neurons. ns, not significant.

In this study, we first attempt to investigate non-monotonic neurons in the ICC using loose-patch recording. Two-hundred and thirteen neurons in the ICC were obtained from 24 mice. When an ICC neuron was loose patched by the pipette, we attempted to obtain the CF of the cell by examining its spike TFR with a tone burst F-A scan. The response-intensity function of the cell was then tested at the CFs. The CFs of the recording neurons were between 4 and 39 kHz (16.0 ± 8.1 kHz) and corresponded to a dorsal-to-ventral (low-to-high) gradient of the CF according to the recording depth (range from 500 to 1,500 μm). The first spike latencies at the HA of the recording neurons range from 8.57 to 78.43 ms (19.61 ± 0.64 ms) and the median is 16.82 ms. Figures 1A,C show the TRFs of two-example ICC neurons with CFs of 16.0 kHz and 13.9 KHz. The first neuron exhibited a typical monotonic response-intensity function (Figure 1B) with a continuously increasing firing rate as the intensity of the CF increased. However, the other neuron did not follow this pattern; its discharge initially increased, peaked at 20 dB SPL, and then decreased despite a continuous increase in sound intensity (Figure 1D). A comparison of these two kinds of neurons shows that the non-monotonic neuron can be viewed as intensity selective, having a preferred intensity and being involved in intensity tuning.

Next, we calculated the MI (spike counts at the HA/that at the BA) to quantify the response-intensity function at the CF. The MI ranged from 0 to 1. A neuron with a smaller MI was considered to be more strongly non-monotonic. The distribution of MI indicates that abundant non-monotonic neurons exist in the ICC with different intensity tuning extents (Figure 1E). A perfect monotonic neuron would generate an MI of 1. According to the criterion from an earlier article (Sivaramakrishnan et al., 2004), we defined non-monotonic neurons as those with an MI < 0.8, saturating neurons as those with 0.8 ≤ MI < 1, and monotonic neurons as those with MI = 1. Our data suggested that approximately 62.9% of the neurons in the ICC exhibited non-monotonic response-intensity functions, similar to previous recordings in other studies (Tang et al., 2008; Grimsley et al., 2013). In addition, both monotonic and non-monotonic neurons had a similar average CF overall (Figure 1F, *t*-test: *t* = 0.619, *df* = 211, *p* = 0.537).

### The Difference Between EPSCs and IPSCs in ICC Neurons

**Figure 2:**
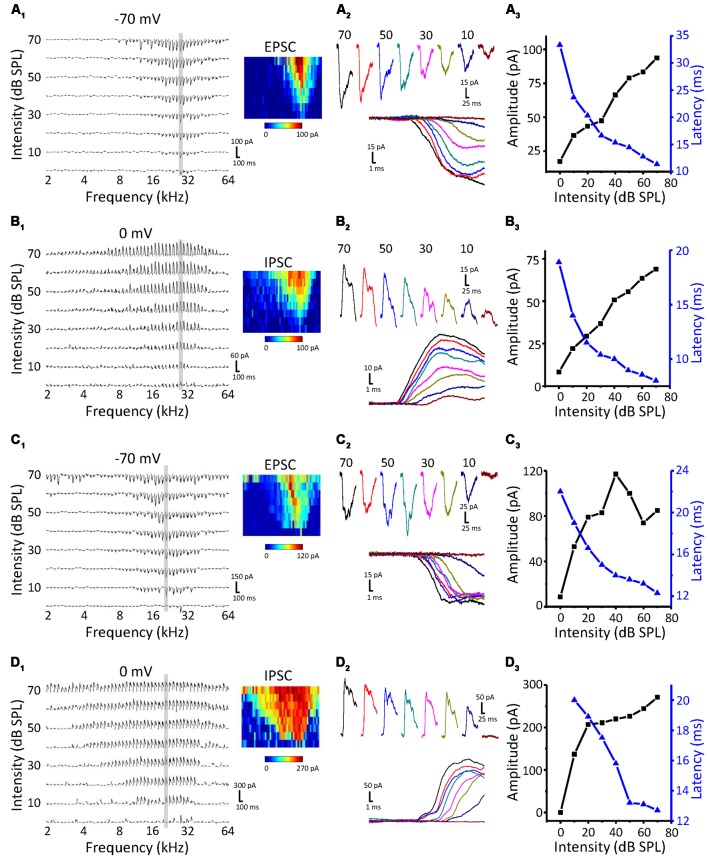
Synaptic inputs to monotonic and non-monotonic neurons in the ICC. **(A,B)** Example excitatory **(A)** and inhibitory **(B)** synaptic inputs recorded in a monotonic neuron. Synaptic TRFs **(A_1_,B_1_)** were obtained with an F-A scan (two repetitions). Each trace in the TRF is the average postsynaptic current response recorded at a particular frequency and intensity. Color represents the amplitude of individual synaptic currents. An enlarged view of the synaptic currents evoked by the CF tone is shown in **(A_2_,B_2_)**, showing the details of the amplitude and latency of synaptic currents. **(A_3_,B_3_)** The plotting of response- (black) and latency-intensity (blue) functions to the synaptic responses at the CF. **(C,D)** Example excitatory **(C)** and inhibitory **(D)** synaptic inputs recorded in a non-monotonic neuron. Data are presented in the same way as in **(A,B)**. Panels **(C_1_,D_1_)** show the synaptic TRFs. **(C_2_,D_2_)** The enlarged view of synaptic currents evoked by the CF tone. **(C_3_,D_3_)** The plotting of response- (black) and latency-intensity (blue) functions to the synaptic responses at the CF.

To study the synaptic inputs of ICC non-monotonic neurons, we used an *in vivo* whole-cell voltage-clamp recording technique. The recording sites were the same as the cell-attached recordings, which were restricted in the ICC region. As expected, we successfully recorded both excitatory postsynaptic currents (EPSCs) and inhibitory postsynaptic currents (IPSCs) from 71 ICC neurons and only obtained the EPSCs from seven neurons and only IPSCs from six neurons. Similar to the extracellular recordings, neurons were first clamped at −70 mV to record EPSCs to obtain the TRF (Figure 2A_1_). The CF of the neuron was usually estimated according to the TRF of the EPSCs because the TRFs of excitatory and inhibitory inputs exhibit the same CF. To characterize synaptic intensity-tuning properties, we randomly interleaved tone bursts at the CF from 0 to 70 dB SPL in 10 dB SPL steps. After recording the synaptic currents at −70 mV, we changed the holding potential to 0 mV to obtain IPSCs (Figure 2B_1_). There are two types of neurons, monotonic and non-monotonic, which can be roughly divided by their excitatory responses (Figures 2A_1_,B_1_ vs. Figures 2C_1_,D_1_). At the CF, both types of neurons exhibited robust synaptic responses (Figures 2A_2_–D_2_) with low thresholds near to 10 dB SPL. Plotting the peak amplitude of synaptic currents clearly showed that the EPSC responses of Neuron 1 gradually strengthened with increasing intensity (Figure 2A_3_, black line), whereas the EPSC responses of Neuron 2 reached a peak at 40 dB SPL and then dropped off, showing a non-monotonic function (Figure 2C_3_, black line). However, the IPSCs of both neurons showed monotonic response-intensity functions (Figures 2B_3_,D_3_, black lines). In addition, both the excitatory and inhibitory response onset latency exhibited monotonic changes with intensity (Figures 2A_3_–D_3_, blue lines).

**Figure 3:**
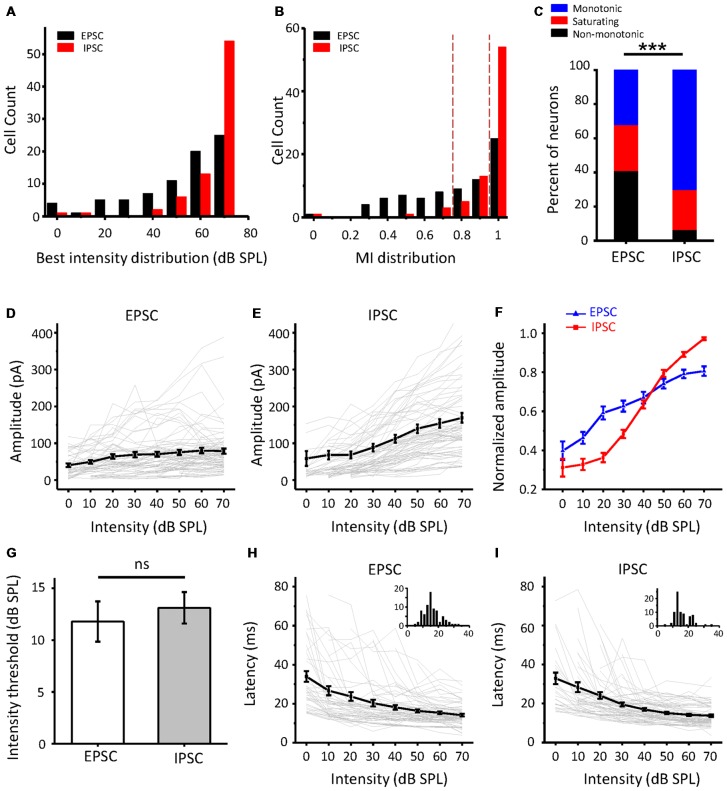
Comparison of excitatory and inhibitory inputs to the ICC in population neurons. **(A)** The distribution of the best intensity of excitatory (black, *n* = 78) and inhibitory (red, *n* = 77) responses, respectively. **(B)** The distribution of the MI of the synaptic responses. **(C)** Bar graph illustrating the percentage of the three types of intensity responses in excitatory postsynaptic currents (EPSCs) and inhibitory postsynaptic currents (IPSCs). ***p < 0.001. **(D,E)** Average of the amplitude response-intensity function at the CF tone for all the neurons’ EPSCs **(D)** and IPSCs **(E)** of the neurons, respectively. The gray lines indicate the individual response-intensity functions. **(F)** Normalized peak current amplitude as a function of sound intensity with all EPSCs and IPSCs in **(D,E)**. **(G)** Comparison of the average synaptic response thresholds between EPSCs and IPSCs. ns, not significant. **(H,I)** Average of the latency-intensity function at the CF for all EPSCs **(H)** and IPSCs **(I)** of the neurons. The gray lines indicate the individual latency-intensity functions. Inserts: the distribution of the onset latencies of synaptic response in EPSC and IPSC, respectively; the abscissa represents time in milliseconds, the ordinate represents cell count.

As the intensity-tuning properties of more neurons were collected, we noticed that there were true differences between EPSCs and IPSCs. As shown in Figure 3A, the best intensity of IPSCs (red column) at the maximal amplitude was mainly concentrated at a higher intensity than that of EPSCs (black column). The MI (Figure 3B) was also obviously different between excitatory and inhibitory inputs; more monotonic response-intensity functions were observed in ICC inhibitory inputs than in excitatory inputs (70.1% vs. 32.1%), while the frequency of non-monotonic inputs in IPSCs was relatively low (6.5%, 5/77). Moreover, the Fisher’s exact test revealed significant differences in the tuning-pattern distribution between the EPSCs and IPSCs (Figure 3C, *χ*^2^ = 30.574, *df* = 2, *p* < 0.001). All synaptic response-intensity functions are plotted in Figures 3D,E for EPSCs and IPSCs, respectively. The average amplitude curves revealed a rising trend with an increase in intensity, although some individual cases deviated from this pattern. Nevertheless, the curves averaged by the normalized peak response amplitude revealed an apparent disparity between excitation and inhibition (Figure 3F). At lower intensities from 0 to 30 dB SPL, the EPSC amplitude rose faster than the IPSC amplitude, but the opposite was true at higher intensities. This observation is mainly due to the contribution of the non-monotonic and monotonic excitatory inputs to the increase in amplitude at lower intensities, whereas the non-monotonic excitatory inputs were weaker at higher intensities. Although the average response threshold of IPSCs was slightly higher than that of EPSCs, this difference was not significant (Figure 3G, *t*-test: *t* = 0.535, *df* = 153, *p* = 0.593). Non-monotonic responses at the CF would seemingly be less likely to be caused by the different thresholds of synaptic inputs. Both the EPSCs and IPSCs showed a monotonic and balanced pattern when latency was plotted (Figures 3H,I). Together, these data suggest that the ICC neurons receive the noticeably unbalanced synaptic inputs and that a certain proportion of EPSPs show non-monotonic amplitude, whereas IPSCs are always monotonic.

### Synaptic Mechanism Underlying Non-monotonic Neurons

**Figure 4:**
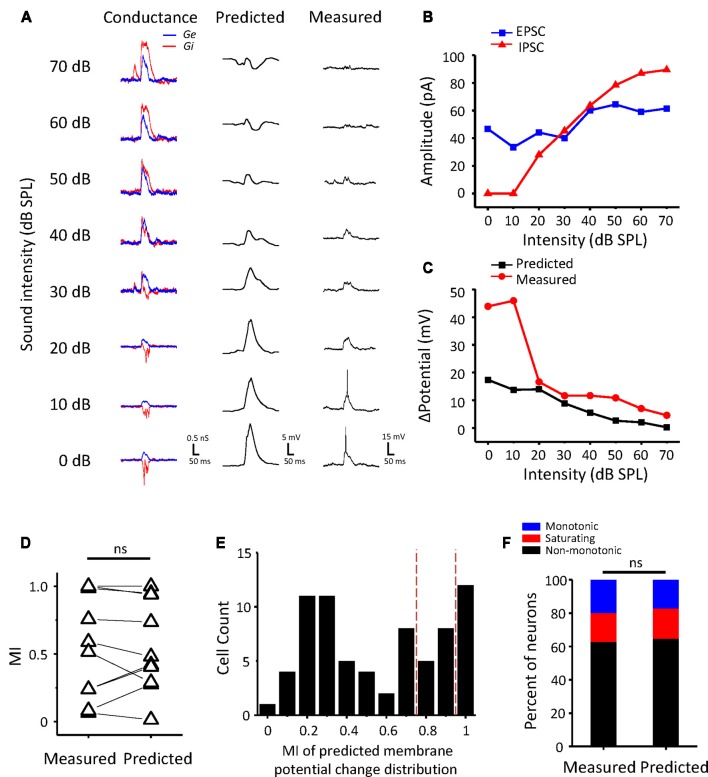
Conductance-based membrane potential predicts the output pattern of ICC neurons. **(A)** The synaptic conductance (left), predicted membrane potential (middle) and recording membrane potential (right) for an example ICC neuron. Excitatory (black) and inhibitory (red) conductance calculated from the current responses. **(B)** The peak amplitude of the EPSCs (black) and IPSCs (red) of the example neuron changed with sound intensity. **(C)** Plotting of the peak membrane potential from the baseline against sound intensity. **(D)** Comparison of MI with measured and predicted peak membrane potential change (*n* = 10). ns, not significant. **(E)** Distribution of the MI of predicted peak membrane potential change in all ICC neurons. The red dashed lines indicate the divide of monotonic, saturating and non-monotonic neurons. **(F)** The proportion of the three types of neurons in two groups of neurons measured in spike counts and estimated by predicted peak membrane potential change, respectively.

**Figure 5:**
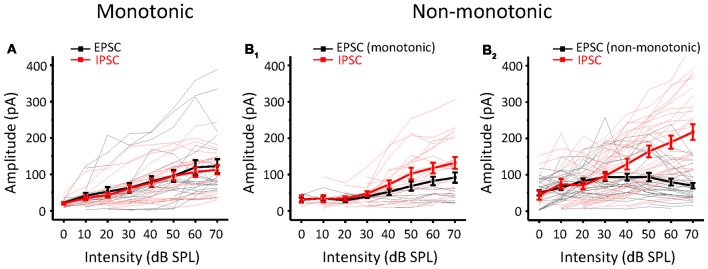
Comparison of EPSCs and IPSCs according to different output patterns. **(A)** The amplitude response-intensity functions for monotonic response (MI ≥ 0.8) neurons’ EPSCs (black lines) and IPSCs (red lines). Thicker lines indicate the average of them. **(B)** The amplitude response-intensity functions for non-monotonic (MI < 0.8) response neurons’ EPSCs and IPSCs. **(B_1_)** The neurons with EPSC MI ≥ 0.8. **(B_2_)** The neurons with EPSC MI < 0.8.

Whether a neuron is monotonic or non-monotonic is decided by its spike discharge rather than its inputs. According to the results in ICC neurons, non-monotonic responses were much less frequent in synaptic inputs than in extracellular recordings. To demonstrate the effect of synaptic inputs on the intensity-tuning profile of output responses, we adopted a conductance-based neuron model for membrane potential estimation (Tan et al., 2007). We resolved the excitatory and inhibitory conductance (Figure 4A, left) from the synaptic current responses and derived the predicted membrane potential (Figure 4A, middle) at different tone intensities following the model. In addition, the intensity tuning of the predicted membrane potential could match well with the measured membrane potential in the practical experiment (Figure 4A, right). As shown in the example neuron, the synaptic amplitude functions could not be classified as non-monotonic (Figure 4B), but their output membrane potential change was typically non-monotonic because the peak of the membrane potential dropped as the intensity increased (Figure 4C). In this manner, we compare another neurons’ MI of between measured and predicted membrane potential (Figure 4D, paired *t*-test: *t* = 0.087, *df* = 9, *p* = 0.933). In all test neurons, the conductance-based neuron model can reflect the practical membrane potential change well. We calculated the predicted membrane potential of all 71 neurons and classified the peak membrane potential tuning curves according to the MI. As shown in Figure 4E, there were more membrane potential tuning curves tending towards non-monotonic when excitatory and inhibitory inputs were integrated. We compared the composition of the intensity-tuning function between membrane potential outputs and the previous spike fired and found no significant differences between these two datasets in the proportion of the three types of neurons (Figure 4F, Chi-Square test: *χ*^2^ = 0.279, *df* = 2, *p* = 0.870). Accordingly, the MI of peak membrane potential was greater than 0.8 or not, we showed the neurons’ EPSCs and IPSCs response as monotonic (Figure 5A) and non-monotonic (Figure 5B) response neurons, respectively. In the monotonic response neurons, their average peak amplitude of EPSCs and IPSCs were balanced with the sound intensity change. However, in the monotonic response neurons, the average amplitude of IPSCs was gradually greater than that of EPSCs after sound intensity higher than 40 dB SPL (Figure 5B_1_), the disparity of amplitude even raise greatly when the EPSCs belonged to non-monotonic itself (Figure 5B_2_). This finding indicated that the unbalanced synaptic inputs could lead to the creation of the non-monotonic neurons in the ICC.

**Figure 6:**
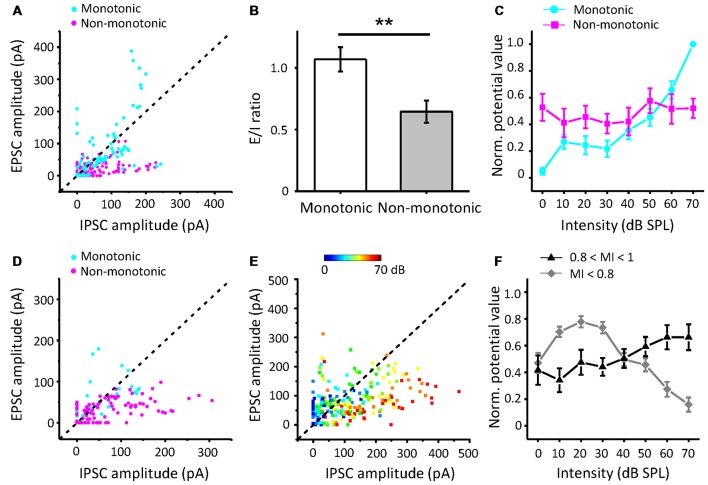
The relative strengths of inhibitory inputs contributing to the generation of non-monotonic neurons. **(A)** Peak EPSC amplitude vs. peak IPSC amplitude evoked by CF tones in neurons with EPSC MI = 1. Blue points represent the predicted membrane potential of neurons (*n* = 10) with monotonic patterns, while magenta points indicate neurons (*n* = 14) in non-monotonic patterns. **(B)** Average excitation/inhibition (E/I) ratios from neurons with EPSC MI = 1 in monotonic and non-monotonic pattern of outputs. **p < 0.005. **(C)** The average normalized peak potential with sound intensity for neurons with EPSC MI = 1. **(D)** Peak EPSC amplitude vs. peak IPSC amplitude in neurons with EPSC 0.8 ≤ MI < 1. **(E)** Peak EPSC amplitude vs. peak IPSC amplitude in neurons with EPSC MI < 0.8. Color represents sound intensity. **(F)** Normalized peak potential with sound intensity for neurons with EPSC 0.8 ≤ MI < 1 and MI < 0.8, respectively.

Furthermore, to study how the excitatory and inhibitory inputs create non-monotonic neurons, we analyzed the excitation/inhibition (E/I) ratios of the recorded neurons in the subgroups sorted by the MI of EPSCs. The peak EPSC amplitude was plotted against the peak IPSC amplitude in neurons whose EPSC MI was equal to 1, as shown in Figure 6A. According to the MI of the membrane potential, we identified the neurons as monotonic (MI = 1) or non-monotonic neurons (MI < 1). The data points for most of the non-monotonic neurons are distributed below the dashed line, where the EPSC amplitude is equal to the IPSC amplitude. In contrast, the points for the monotonic neurons are near or above the dashed line. When the E/I ratio of a neuron is equal to or greater than 1, there is a high probability of generating more spikes. For the neurons with EPSC MI = 1, the E/I ratios of monotonic neurons were distinct from those of non-monotonic neurons (Figure 6B, *t*-test: *t* = 3.166, *df* = 142, *p* = 0.002). The stronger inhibition suppresses the generation of spikes, especially at high intensities, because the inhibitory inputs are usually monotonic. Thus, the average curve of normalized peak amplitudes of membrane potential responses approximates a horizontal line with the changing intensity (Figure 6C, magenta line).

For the neurons with EPSC MI greater than 0.8 and less than 1 (Figure 6D), most of the E/I ratios were less than 1, indicative of predominant inhibitory inputs. Although the excitatory inputs of these neurons were considered to be saturated, the continuously increasing inhibition shaped their output responses to be more non-monotonic, as the mean MI membrane potential response reduced to 0.66. However, in some cases, the neurons (*n* = 4) received relatively weak inhibition, and hence, their outputs inherited the excitatory inputs (Figure 6D, cyan points).

For the neurons with EPSC MI less than 0.8 (Figure 6E), the E/I ratios are seemingly well distributed along the dashed line. When we identified the E/I ratios with the sound stimuli intensities, we found that the EPSC amplitudes were generally greater than IPSC amplitudes at lower intensities (Figure 6E, cool-colored points), and at higher intensities, the IPSC amplitudes were generally greater than the EPSC amplitudes (warm-colored points). This observation suggests that the neurons with afferent non-monotonic EPSCs would be more easily affected by the inhibition and generate typical non-monotonic outputs heavily inhibited at the high intensities but discharging at other lower intensities. Consistent with this finding, the average curve of normalized membrane potential values (Figure 6F, gray line) clarifies the summation of non-monotonic neurons responses in which the response is larger at lower intensities and gradually decreases at higher intensities. Thus, the integration of non-monotonic EPSC and monotonic IPSC responses could shape the non-monotonic neurons in the ICC.

## Discussion

**Figure 7:**
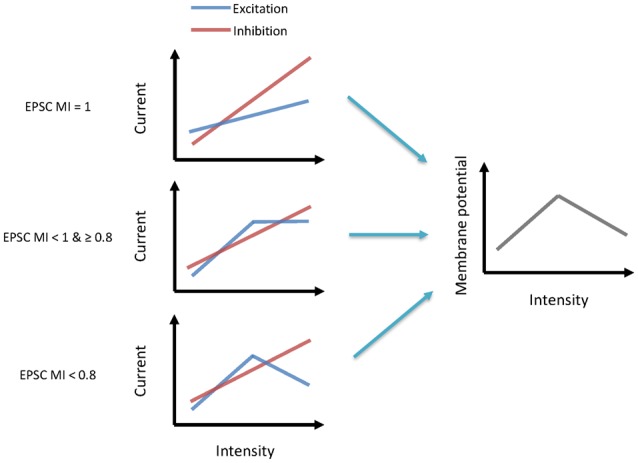
Synaptic mechanisms underlying the generation of non-monotonic neurons. Left schematics indicate the excitatory and inhibitory current inputs to neurons from low to high-intensity sound (from left to right). Each row illustrates a different pattern. Right schematics indicate their output membrane potential.

The main goal of our study was to explore the underlying synaptic mechanisms in the generation of non-monotonic neurons in the ICC. By obtaining the synaptic inputs using our voltage-clamp whole-cell recordings in ICC non-monotonic neurons, we found differences in the intensity-tuning properties of excitatory and inhibitory inputs; 41% of neurons received non-monotonic excitation, whereas their inhibitory inputs were monotonic. Interestingly, the proportion of non-monotonic synaptic inputs (Figure 1E) was smaller than that of non-monotonic neuronal outputs (Figure 3B), indicating that the non-monotonic properties of the neurons of the ICC are not completely inherited from the lower nuclei and that the IC can also produce non-monotonic neurons *de novo*. Furthermore, by using a membrane conductance model to predict membrane potential output and analyzing the ratios of excitatory and inhibitory responses (Figure 4), we concluded a general rule that a relatively strong and monotonic inhibitory input is the key to generating non-monotonic properties in neurons in the ICC (Figures 5, 6). To better understand how the synaptic inputs contribute to the generation of non-monotonic neurons, we summarized the probable mechanisms in the schematic shown in Figure 6. When the intensity of excitatory inputs to a cell are monotonically increasing at a slower pace than that of inhibitory inputs (Figure 7, first row), the final output response-intensity function of the cell may be non-monotonic. When the excitatory input is initially monotonic but saturated after a certain intensity (Figure 7, middle row), the output response of the cell may also become non-monotonic due to the continuous increase in inhibitory input. When the excitatory input is inherently non-monotonic (Figure 7, bottom row), the non-monotonic output response of the cell will become more prominent due to the inhibitory input. Taken together, these observations indicate that the monotonic increase in the inhibitory input in the ICC is likely to be involved in generating non-monotonic outputs.

The phenomenon of non-monotonic neurons has been extensively studied since their discovery. Previous researchers have investigated with local injection (Yang et al., 1992; LeBeau et al., 2001; Sivaramakrishnan et al., 2004; Tang et al., 2008; Grimsley et al., 2013), two-tone masking (Jen and Wu, 2005; Egorova and Ehret, 2008), etc., suggesting that the IC can create non-monotonic neurons by itself. However, these studies all used extracellular recordings, and there was no direct detection of an inhibitory response to cells. We were the first to directly examine the synaptic inputs for ICC non-monotonic neurons. The proportions of non-monotonic neurons in the IC are not uniform among studies, mainly due to the different definitions of non-monotonic neurons and experimental methods used. After excluding the effects of these interfering factors, our data agree with the previous reports. Indeed, there are a large number of non-monotonic neurons in the ICC (Figure 1).

In our study, we concluded that the differences in the intensity response between EPSCs and IPSCs were the cause of the generation of non-monotonic neurons (Figure 6). The most dramatic distinction these two types of synaptic inputs was their unbalanced proportion of intensity-tuning properties. Why the IPSCs that ICC neurons received were always responses of monotonic intensity and the EPSCs were not was an interesting question (Figure 3). To answer this question, the regular anatomical connectivity of the ICC needs to be examined. The ICC combines abundant binaural and monaural source auditory information from almost all auditory brainstem nuclei (Grothe et al., 2010; Ito et al., 2016). The ICC also accepts reciprocal projections from the contralateral IC and downward projections from the AC and MGB (Lee et al., 2015; Patel et al., 2017; Clarke and Lee, 2018). The excitatory inputs to the ICC mainly come from the contralateral CN, lateral superior olive (LSO), bilateral intermediate nucleus of the lateral lemniscus (NLL), and the ipsilateral medial superior olive (MSO; Loftus et al., 2004; Ito et al., 2011). The inhibitory inputs come from the ascending innervation that begins at the ipsilateral LSO, ventral NLL, and bilateral dorsal NLL (Bajo et al., 1998; Batra and Fitzpatrick, 2002), as well as directly from local interneurons (Schofield and Beebe, 2018). The different sources of inhibition may contribute to the discrepancies in EPSCs and IPSCs. Thus, non-monotonic neurons were thought to result from their high-threshold and intensity-dependent inhibitory inputs. In other words, the unbalanced thresholds between excitatory and inhibitory inputs generate non-monotonic neurons. However, our data suggest that thresholds for excitatory and inhibitory responses are not significantly different in CF conditions (Figure 3G), which is in line with previous reports (Ono and Oliver, 2014b; Wei et al., 2018). Additionally, using an optogenetic method, researchers successfully identified GABAergic neurons in the IC *in vivo* (Ono et al., 2017). Comparing the responses properties of GABAergic and glutamatergic neurons, they found that there were no differences in spike thresholds, latencies, or rate-level functions overall. The ICC circuit alone does not seem to be sufficient to produce a gap between excitation and inhibition. All IC neurons receive parallel excitatory and inhibitory inputs from the lower auditory brainstem that provide temporally synchronous balanced synaptic inputs. Therefore, this interpretation of threshold difference between excitation and inhibition cannot apply to the ICC, at least, not all ICC neurons. A previous study showed that in the AC, changes in the relative time interval between excitation and inhibition could also create non-monotonic tuning (Wu et al., 2006). Is there a similar change in the ICC? According to our experimental results, the inhibition delay may have a small effect; however, the different latencies between EPSCs and IPSCs are not obvious at the CF (Figures 3H,I), are much shorter than the duration of the response of ICC neurons and not enough to produce most of the discharge. Then, we derived another possible explanation for the monotonic IPSCs. The direct ascending inhibition provides initial inputs and determines the threshold and CF of the IPSC. Additionally, the local inhibition, especially at higher intensities, maintains the increase in the responses, thereby making the majority of IPSCs monotonic. To prove this hypothesis, additional studies of synaptic input sources should be further identified. It is also important to note that in this study, due to technical reasons, recordings were performed in anesthesia, which affects neuronal responses in IC by enhancing the inhibition (Kuwada et al., 1989). In pharmacological experiments, the pentobarbital is demonstrated to shift the dose-response curve with GABA concentration, while at a high level of GABA the change of peak Cl^−^ currents was not evident (Parker et al., 1986; Franks and Lieb, 1994). Thus, it may prolong the duration of inhibition but not affect the rise time and peak amplitude (Gage and Robertson, 1985; MacIver et al., 1991; Franks and Lieb, 1994), and unlikely to alter the monotonic properties of inhibition. It has also been reported that, in the extracellular recordings, the neurons’ spontaneous activity, sound-evoked firing pattern, minimum threshold and latency were significantly changed in pentobarbital-anesthetized animals (Kuwada et al., 1989; Fitzpatrick et al., 2000; Tan et al., 2008; Liang et al., 2011). Although the cell-attached and whole-cell recordings were conducted under the same anesthetic, recordings for awake animal will be better to reveal the role of inhibition in the non-monotonic neurons.

In this study, we used the 50-ms tone burst at contralateral stimuli only, thus how the non-monotonic synaptic behavior under binaural condition remained uncertain. The IC is a binaural structure. For sound localization in small animals, the cue of interaural level difference (ILD) plays an important role. A recent *in vivo* whole-cell study in mouse IC revealed that there are three types (contra-preferred, U-shaped and center-preferred) of synaptic responses of neurons to constant average binaural level (ABL) stimuli (Ono and Oliver, 2014b). The ABL methods, in which the intensity of contralateral and ipsilateral stimuli increase or decline in opposite directions, were usually used to simulate sound source at different azimuth by ILD changing from −40 to 40 dB (Irvine and Gago, 1990; Greene et al., 2014). According to Ono and Oliver’s article, in the contra-preferred and the U-shaped types, there are excitatory and inhibitory synaptic inputs balance in changing with the different ILD. In both patterns of neurons when the contralateral stimuli are higher and the ILD is negative, the peak amplitude and charge were monotonically increasing, suggesting that the monotonic neurons in our study may belong to the two types. The different monotonically changing in synaptic responses at positive ILDs between them may come from the stronger responses to ipsilateral stimuli in the U-shaped neurons. For the center-preferred type neurons, EPSCs showed more non-monotonic functions with ILDs and contralateral level changes, while the IPSCs were not. This finding is consistent with our observation in non-monotonic neurons. Although the result of small samples (5/30) in their article deviate from ours, we still expect our conclusion about the synaptic inputs underlying non-monotonic neurons to also hold in the binaural condition since the contralateral stimuli are prevailing in binaural responses (Xiong et al., 2013; Wei et al., 2018) and there is a high proportion of non-monotonic neurons at binaural stimuli (Irvine and Gago, 1990; Aitkin, 1991; Tan et al., 2008; Grimsley et al., 2013). Another work of Ono and Ono and Oliver (2014a) by using *in vivo* whole-cell technology shows that the sound evoked EPSCs have more variability and the shortest peak latencies in mouse ICC neurons. However, the peak times of synaptic currents in our recordings range from 10 to 100 ms and most of the peak times concentrated below 40 ms at the HA (data not shown). Thus, the built-up type of EPSCs they described was not found in our study. The difference may be due to the duration of sound stimuli, which we used is shorter than that they used (200 ms). Nevertheless, our results support their idea about the level invariance of peak time that most of the coefficients of variance of peak times across different sound intensities were lower than 0.2. It’s suggested that, for most neurons, the temporal property is a fixed property of synapse. Moreover, the temporal properties of excitatory and inhibitory synaptic inputs change in balance. So a few milliseconds discrepancy of peak times and durations may have less effect on the monotonic properties. According to their integrate-and-fire model, the diverse firing patterns mainly come from different neurons. Since the duration of sound stimuli may affect the temporal properties of synaptic responses, it still needs further study to explore the temporal properties with the different duration and SPL stimuli.

In this study, we used the peak membrane potential as an indicator of cellular output (Figures 4–6). In fact, the peak membrane potential is not equivalent to the spike rate. As we know that ICC neurons have many diverse types of neurons, i.e., sustained, adapting and onset cell, dividing by their fire patterns with the injection of depolarizing current (Bal et al., 2002; Yassin et al., 2016). Due to their distinct intrinsic membrane properties, the application of the same stimulation to different types of neurons can drive different discharge responses. However, for almost all types of ICC neurons, their firing rates would change with the injection current change (Tan and Borst, 2007; Xie et al., 2008; Yassin et al., 2016), except the onset\transient neuron, which only generate a single spike at initiation (Tan and Borst, 2007; Yassin et al., 2016). As a result, the spike rate can be modeled as a threshold-monotonic function of the membrane potential. The difference among these neurons is their dynamic range of spike rate change across the different intensity stimuli. What’s more, sustained cell, the most common type in the ICC, has the largest dynamic range and lineally function between fire rate and injected current (Xie et al., 2008; Liu et al., 2015). Only less than 5% of neurons showing an onset and transient fire pattern are not sensitive to the excitatory postsynaptic potential change (Liu et al., 2015). It is reasonable for spike threshold sharpens intensity tuning similarly to it sharpens frequency tuning (Tan et al., 2007). Thus, the peak membrane potential can reflect its spike rate and may be even more sensitive in the prediction of non-monotonic properties.

Although not the focus of this study, the functional perspective of non-monotonic neurons in the ICC is also interesting. Recently, studies in the marmoset primary AC indicated that non-monotonic neurons may complement the function of monotonic neurons in different sound-encoding contexts (Sadagopan and Wang, 2008; Sun et al., 2017). In these views, the non-monotonic neurons in the IC may be beneficial to the concentration of non-monotonic neurons in the AC. According to differences in TRF curves, there are typically three types of neurons, type V, type I and type O neurons, which can also be found in the AC and in the ICC (Jen and Zhang, 2000; LeBeau et al., 2001; Barbour, 2011). Type I neurons are formed from type V neurons by a sharpening of the bandwidth; moreover, the TRF curve of type O neurons is shaped like that of type I neurons, with rates decreasing at a higher intensity sound. The change in these three types of neurons is indicative of the gradual strengthening of central inhibitory processes. The multistage transmission of the auditory system extracts the frequency and intensity information, improved resolution (Suga, 1995) and is helpful for the dynamic range of the intensity coding, enhancing sensitivity to soft sounds and protecting against loud noise. Moreover, because the IC has abundant connections with the other auditory areas and integrates binaural acoustic information, it plays a critical role in processing spatial hearing. The intensity differences between the two ears are important for analyzing the sound source. How non-monotonic neurons perform in binaural integration and sound localization remains an interesting issue for further research.

In conclusion, by measuring the synaptic inputs in many ICC neurons, we demonstrated that non-monotonic intensity responses can be created or shaped by the inhibition, which is much more dependent on the relative E/I amplitude interaction than on thresholds and latencies of E/I.

## Data Availability

All datasets generated for this study are included in the manuscript.

## Ethics Statement

This study was carried out in accordance with the recommendations of guidelines for the Care and Use of Laboratory Animals in Southern Medical University established by the Animal Care and Use Committee of Southern Medical University. All the experiment protocols were approved by the Animal Care and Use Committee of Southern Medical University.

## Author Contributions

ZX and JW conceived and designed the experiments. YL, GZ, JW, HY and HL collected and analyzed data. YL, JW and GZ prepared figures. ZX and YL wrote the manuscript. All authors revised the manuscript critically for intellectual content. All authors have approved the final version of the manuscript and agree to be accountable for all aspects of the work.

## Conflict of Interest Statement

The authors declare that the research was conducted in the absence of any commercial or financial relationships that could be construed as a potential conflict of interest.
